# OS-DETR: End-to-end brain tumor detection framework based on orthogonal channel shuffle networks

**DOI:** 10.1371/journal.pone.0320757

**Published:** 2025-05-13

**Authors:** Kaixin Deng, Quan Wen, Fan Yang, Hang Ouyang, Zhuohang Shi, Shiyu Shuai, Zhaowang Wu

**Affiliations:** College of Computer Science and Cyber Security, Chengdu University of Technology, Chengdu, China; Fudan University, CHINA

## Abstract

OrthoNets use the Gram-Schmidt process to achieve orthogonality among filters but do not impose constraints on the internal orthogonality of individual filters. To reduce the risk of overfitting, especially in scenarios with limited data such as medical image, this study explores an enhanced network that ensures the internal orthogonality within individual filters, named the **O**rthogonal Channel **S**huffle **Net**work ( **OSNet**). This network is integrated into the Detection Transformer (DETR) framework for brain tumor detection, resulting in the **OS-DETR**. To further optimize model performance, this study also incorporates deformable attention mechanisms and an Intersection over Union strategy that emphasizes the internal region influence of bounding boxes and the corner distance disparity. Experimental results on the Br35H brain tumor dataset demonstrate the significant advantages of OS-DETR over mainstream object detection frameworks. Specifically, OS-DETR achieves a Precision of 95.0%, Recall of 94.2%, mAP@50 of 95.7%, and mAP@50:95 of 74.2%. The code implementation and experimental results are available at https://github.com/dkx2077/OS-DETR.git.

## Introduction

In recent years, significant advancements in medical technology have greatly contributed to brain tumor research and treatment, deepening our understanding of their molecular and structural characteristics. Among these technologies, gene sequencing plays a pivotal role by uncovering the molecular biology underlying brain tumors and enabling the development of personalized treatments [1]. High-quality imaging diagnostics, including skull CT [2] and MRI [3], are essential for the accurate detection and quantitative evaluation of brain tumors. These imaging tools provide comprehensive lesion visualization, empowering physicians to make informed clinical decisions and improve patient outcomes. Despite these advancements, there remains substantial potential for further innovation, particularly in applying object detection techniques to enhance diagnostic efficiency.

Object detection, a fundamental area in computer vision, is widely applied to identify and localize object instances within predefined categories. Traditional object detection often relies on the careful design and extraction of manual features [4, 5]. However, the development of deep learning, particularly convolutional neural networks (CNN) [6, 7], has revolutionized this field, enabling more robust and accurate detection. Modern deep learning-based object detection approaches are typically categorized into one-stage and two-stage methods [8]. In one-stage methods, the most influential framework is You Only Look Once (YOLO). With its efficient feature extraction capabilities, YOLO has set a benchmark for real-time object detection tasks and is widely applied across various fields [9, 10]. These applications highlight its adaptability in addressing complex challenges. The introduction of DETR [11] marks a milestone in object detection, framing it as a set prediction problem. Leveraging the Transformer’s sequence transformation capabilities [12], DETR directly converts image sequences into set sequences. This eliminates the need for region proposal networks and anchor mechanisms, enabling an end-to-end detection framework.

**Fig 1:**
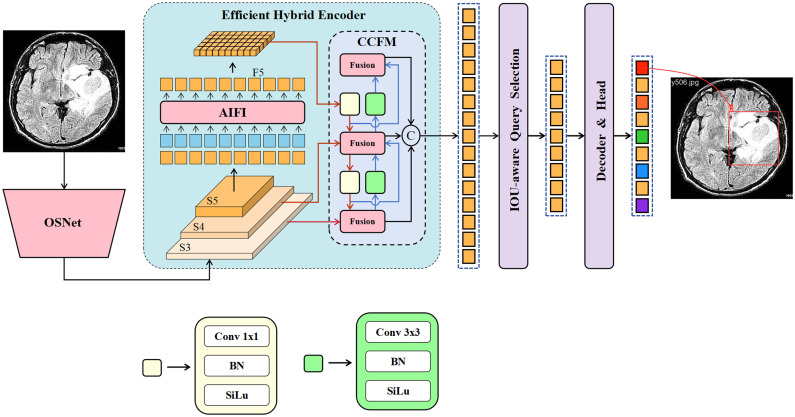
The architecture of the OS-DETR. Compared to RT-DETR, this study introduces several improvements to the network architecture. First, OS-DETR incorporates the OSNet into the backbone network to enhance feature extraction capabilities. Second, OS-DETR optimizes the encoder part to strengthen the fusion of cross-scale information.

However, within medical image, the majority of studies have focused on classification [13] and segmentation [14, 15], with relatively few studies on brain tumor object detection. Currently, most existing approaches are the YOLO-based framework [16–19] in the medical image, while research exploring end-to-end object detection framework remains limited. DETR has demonstrated highly competitive performance on the large-scale COCO [20] dataset. However, Research [21] shows a significant decline in its performance when applied to small-scale datasets, indicating a strong dependency on data quantity. To address this issue, this study introduces a simple and efficient feature extraction network as the backbone of the detection framework. This network reduces the risk of overfitting and exhibits notable advantages, particularly in the field of medical imaging, where data availability is often limited. Specifically, this study explores an enhanced model, the OS-DETR, as depicted in Fig 1. Similar to other DETR-based frameworks, OS-DETR consists of a backbone network, an improved efficient encoder, and a decoder.

The primary contributions in this study are as follows:

1. This study introduces the OSNet, which utilizes the Special Orthogonal Group process to initialize filters, ensuring orthogonality within their internal spatial structure.
2. In the encoder component, OS-DETR incorporates the deformable attention mechanism to replace the multi-head self-attention mechanism commonly used in other DETR-based framework.
3. OS-DETR incorporates ICAD-IoU, which integrates the influence of internal regions with corner distance discrepancies.

The structure of this paper is organized as follows: Related Work section provides a comprehensive review of mainstream object detection algorithms and analyzes the literature focusing on algorithms designed for brain tumor detection, systematically outlining the latest progress and findings in the field. Materials and Methods section details the research methods employed in this study, including the implementation specifics, key techniques, and innovative approaches. Experiments and Results section presents the experimental setup and environment, reports the results of comparative and ablation studies, and conducts an analysis of the findings. Finally, Discussion and Conclusion section summarizes the primary content and conclusions of the study while proposing future research directions and potential improvements.

## Related work

### You Only Look Once (YOLO)

Real-time object detection technology is necessary in various fields. The YOLO framework stands out among object detection algorithms for its ability to accurately complete detection tasks in a single pass, positioning it as a mainstream option. Originally proposed by Joseph Redmon [22], YOLO differs from prior methods in that it does not require sliding windows, the execution of numerous classifiers per image, or a two-stage approach involving the detection of potential object-containing areas followed by classification. Instead, YOLO utilizes a straightforward regression output for prediction, unlike Fast R-CNN [23], which employs dual independent outputs for classification probability and bounding box coordinate regression.

The YOLO series consistently aims to balance speed with accuracy for real-time performance. However, the initial model compromised some accuracy, especially with small objects or overlapping boxes, in pursuit of high-speed detection. As YOLO evolved, striking a balance between speed and accuracy became a focal point. With the integration of anchor boxes and transfer layers in YOLOv2 [24], the accuracy of object localization improved. YOLOv3 [25] adopted a multi-scale feature extraction architecture, enhancing adaptability to objects of varying scales. As the framework progressed, achieving a nuanced balance between speed and accuracy emerged as a key challenge. YOLOv4 [26] and YOLOv5 introduced novel network backbones, enhanced data augmentation methods, and refined training strategies, significantly boosting accuracy while retaining real-time performance.

YOLOv7 [27] introduces the Extended Efficient Layer Aggregation Network (E-ELAN), a strategy that enables deep models to learn and converge more efficiently by managing the shortest and longest gradient paths. E-ELAN enhances the network’s learning capability without disrupting the original gradient paths through shuffling and merging bases to combine features from different groups. This approach is particularly effective for models with infinite stacks of computational blocks. The architecture of YOLOv7 is cascade-based, and when standard scaling techniques like depth scaling are applied, a transition layer is created. YOLOv8 represents an advanced model for object detection and semantic segmentation. It introduces a redesigned Cross Stage Partial Layer (CSPLayer) [28], referred to as the Cross Stage Partial Bottleneck with two Convolutions. This model employs a decoupled anchorless head to address likelihood, classification, and regression tasks. Additionally, it incorporates Complete Intersection over Union [29], Distribution Focal Loss [30], and Binary Cross-Entropy loss functions.

YOLOv9 [31] overcomes the challenges of information bottlenecks and invertible function limitations in deep networks by introducing two novel techniques: Programmable Gradient Information (PGI) and the Generalized Efficient Layer Aggregation Network (GELAN). These advancements achieve higher parameter efficiency while relying solely on traditional convolutional operators. YOLOv10 [32] introduces consistent dual allocation, enabling end-to-end training without the need for Non-Maximal Suppression (NMS). The model optimizes each component of YOLO using a comprehensive efficiency-accuracy-driven design strategy. YOLO11 [33] enhances feature extraction capabilities and improves object detection accuracy through its optimized backbone network and neck structure. Additionally, the refined architectural design and streamlined training processes increase processing speed while maintaining a strong balance between accuracy and performance. On the COCO dataset, YOLO11m achieves a higher mean Average Precision compared to YOLOv8m, reducing the number of parameters by 22% and significantly boosting computational efficiency.

### Detection Transformer (DETR)

In recent times, the Transformer has seen widespread application in computer vision’s object classification domain, as evidenced by the Vision Transformer (ViT) and others [34–37]. DETR marks the first use of the classic Encoder-Decoder structure of Transformer in object detection. DETR’s backbone network employs a convolutional network, while its Encoder and Decoder are based on the Transformer framework. Its output layer is a Multi-Layer Perceptron (MLP), and the model utilizes a loss function derived from bipartite graph matching, forming a graph by aligning the ground truth with predicted bounding boxes. DETR comprises four key modules: the backbone, encoder, decoder, and prediction head.

Nevertheless, the initial DETR model faced two major challenges: a slow convergence rate, largely due to the uniform self-attention across each position, requiring more time to identify sparse yet crucial spots; and subpar small object performance, attributed to the absence of a multi-scale structure akin to Feature Pyramid Network (FPN) and the self-attention’s computational complexity being square proportional to the feature map’s area. Addressing these issues, Deformable DETR [38] introduced Deformable Attention to tackle the $\text{O}({\text{N}}^{2})$ problem inherent in standard Transformer’s Attention, thereby hastening model convergence, reducing algorithmic complexity, and incorporating multi-scale features to enhance small object detection efficiency.

However, the DETR series models’ high computational demands constrain their practical application effectiveness and hinder full utilization of post-processing-free benefits, like NMS. To tackle these challenges, Wenyu Lv and colleagues introduced the first real-time, end-to-end object detection framework, Real-time Detection Transformer (RT-DETR) [39]. RT-DETR features an efficient hybrid encoder adept at handling multi-scale features by segregating intra-scale interaction from cross-scale fusion. It also introduces IoU-aware query selection, further enhancing performance by supplying the decoder with superior initial object queries. Additionally, RT-DETR’s capability to adjust inference speed through varying decoder layers, without necessitating retraining, broadens its applicability in diverse real-time contexts. Notably, RT-DETR was the first DETR-based framework that surpassed contemporary YOLO-based framework of comparable scale in both detection speed and accuracy.

### Applications of object detection in brain tumor

RCS-YOLO [17] is an innovative design that achieves an effective balance between feature cascading and computational efficiency. It integrates Reparameterized Convolution based on Channel Shuffle (RCS) and One-Shot Aggregation of RCS (RCS-OSA) methods, which significantly enhance information extraction while minimizing time consumption. The RCS method is inspired by ShuffleNet [40, 41] and leverages channel shuffle to deeply fuse input features across different channels. This approach optimizes the model’s performance in both training and inference phases.

BGF-YOLO [16] is an advanced architecture based on YOLOv8, specifically designed for brain tumor detection. It integrates three key enhancements: Bi-level Routing Attention (BRA) [42], Generalized Feature Pyramid Network (GFPN), and an additional fourth detecting head. BRA employs a dynamic sparse attention mechanism that selectively focuses on salient regions, effectively reducing feature redundancy and improving detection precision. GFPN enables multilevel feature fusion by incorporating dense connections and skip pathways, ensuring robust handling of both high-level semantic and low-level spatial information. The inclusion of the fourth detecting head extends the detection capacity to finer scales, addressing variability in tumor sizes and improving accuracy for larger objects.

## Materials and methods

### Feature extraction network for OS-DETR model

#### Orthogonal Channel Shuffle Network (OSNet).

The concept of channel attention was initially introduced by Squeeze-and-Excitation Networks (SENet) [43]. SENet employs Global Average Pooling (GAP) to reduce the spatial dimension of each feature channel to a single scalar. However, a drawback of SENet is its sole reliance on GAP for channel compression. Addressing this limitation, Frequency Channel Attention Networks (FcaNet) [44] posited that GAP might neglect vital low-frequency information. FcaNet emphasizes the importance of frequency selection in Discrete Cosine Transforms (DCTs) for enhanced information provision. The success of FcaNet is largely due to the orthogonality in DCT compression mapping. Building upon this, Hadi Salman *et al*. developed a channel attention mechanism that utilizes random orthogonal filters to compress spatial information of each feature [45], termed Orthogonal Channel Attention. OrthoNets demonstrated notable results on the ImageNet dataset and achieved superior performance on the Birds and Places365 datasets.

**Fig 2:**
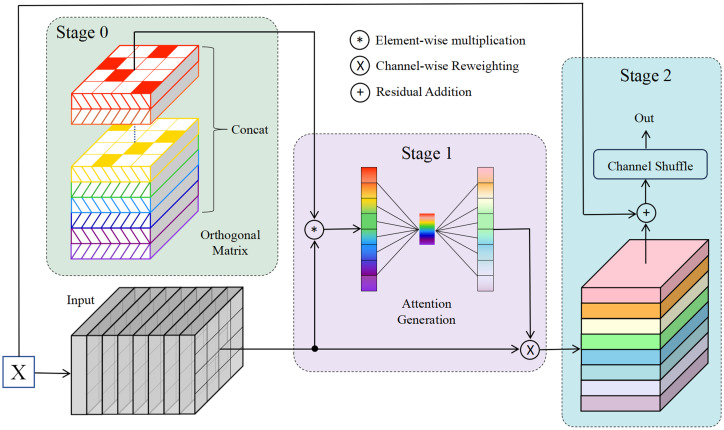
The structure of the OSNet Block. The proposed method consists of three stages. Stage 0: Orthogonal matrices are sampled from the Special Orthogonal Group (SO) to construct orthogonal filters; Stage 1: These orthogonal filters are applied to the input feature maps, followed by dimensionality reduction along the spatial dimensions. This process generates channel attention weights, which are further refined through a fully connected layer and subsequently used to reweight the channels of the original feature map; Stage 2: The channels of the output feature map are then rearranged.

In OrthoNets, the Gram-Schmidt process flattens randomly generated filters into vectors and ensures pairwise orthogonality among these vectors, achieving overall orthogonality between filters. However, this approach does not explicitly constrain the internal orthogonality of individual filters, meaning the orthogonality between rows or columns within each h×w filter is not guaranteed. Constraining internal orthogonality within individual filters can effectively reduce the risk of overfitting, particularly in the medical image field where data is often limited. To address this, this study introduces the OSNet that adopts the special orthogonal group method to initialize filters, ensuring orthogonality within the spatial structure of each filter. This approach guarantees the independence of features within each filter, such as orthogonality among row or column vectors, without imposing orthogonality constraints between different filters, which are independently sampled. The structure of the OSNet Block is shown in Fig 2.

The OSNet Block is designed based on the ResNet architecture and comprises two primary branches: a shortcut branch and a main branch. The shortcut branch adjusts spatial dimensions and channel numbers to match the main branch output. The main branch consists of two 3×3 convolutional layers. The first layer adjusts the spatial dimensions of the input feature map **X**, while the second layer extracts features and outputs the feature map **F**. For the feature map **F** with dimensions (*b*, *c*, *h*, *w*), orthogonal matrices $\{{𝐖}_{i}^{h\times w}{\}}_{i=1}^{c}$ are randomly sampled from the Special Orthogonal Group SO(n), since only square matrices can be ortho gonal matrices, where *n* = *h* = *w*. For each channel *i* (out of a total of *c* channels), an n×n orthogonal matrix ${𝐖}_{i}^{h\times w}\in SO(n)$ is sampled and used as the filter weights. These filters $\{{𝐖}_{i}^{h\times w}{\}}_{i=1}^{c}$ maintain orthogonality within the spatial dimensions, ensuring diversity and independence in the feature extraction process.

All filters $\{{𝐖}_{i}^{h\times w}{\}}_{i=1}^{c}$ are concatenated along the channel dimension, resulting in a combined filter ${𝐖}_{c\times h\times w}$ with dimensions (*c*, *h*, *w*). Element-wise multiplication is performed between the feature map **F** and the corresponding filter weights ${𝐖}_{i}^{h\times w}$ for each channel *i*, preserving the original dimensions (*b*, *c*, *h*, *w*) of the feature map **F**. This operation effectively applies orthogonal constraints to the feature map **F**. A summation operation is then applied across the spatial dimensions (height and width). This operation compresses the spatial information of each channel into a tensor **f** with dimensions (*b*, *c*, 1, 1). This feature transformation projects the input feature map **F** onto the orthogonal subspace represented by the concatenated orthogonal filter ${𝐖}_{c\times h\times w}$. The tensor value of each channel in **f** represents its projection coefficient in the orthogonal subspace. The orthogonal filter ${𝐖}_{c\times h\times w}$, satisfying orthogonal constraints, enables this projection to extract components of feature map **F** along different orthogonal directions. This process reduces redundancy and correlation between features, enhancing the independence and diversity of feature representations.

The orthogonal constraint promotes feature decoupling and diverse representations, allowing the network to better capture the essential structures and characteristics of input data. The resulting tensors **f** undergo a subnetwork comprising two fully connected layers with a ReLU activation and a Sigmoid activation function, producing channel attention weights α with dimensions (*b*,*c*), representing importance weights for each channel. The attention weights α are applied channel-wise to the original feature map **F**, reweighting the importance of each channel. This attention mechanism enables the network to dynamically focus on relevant features, enhancing its ability to capture critical information across different channels.

The attention weights α are reshaped into the dimensions (*b*, *c*, 1, 1) to align with the structure required for element-wise multiplication. These weights are then applied to each channel of the original feature map **F**, producing the reweighted feature map ${𝐅}_{\text{attn}}$. This operation highlights key feature channels while reducing the influence of less important ones, thereby enhancing the network’s ability to focus on essential information within the input data. The reweighted feature map ${𝐅}_{\text{attn}}$ is then combined with the residual connection ${𝐅}_{\text{res}}$ and activated using the ReLU function, resulting in the final feature map ${𝐅}_{\text{out}}$. This step merges the attention-learned feature map ${𝐅}_{\text{attn}}$ with the original input features through the residual connection ${𝐅}_{\text{res}}$, preserving input information while enhancing the feature representation capacity. Subsequently, a Channel Shuffle operation is applied to the merged feature map ${𝐅}_{\text{out}}$. This channel rearrangement promotes information exchange and feature fusion between different channel groups, thereby improving feature complementarity and diversity. Finally, a non-linear activation function, such as ReLU, is applied to the merged feature map ${𝐅}_{\text{out}}$. This step increases the network’s expressive power, allowing it to capture more complex patterns and relationships within the data.

In OS-DETR, OSNet Blocks are stacked, enabling the network to progressively refine feature representations and capture hierarchical patterns. Compared to the Gram-Schmidt method employed in the original OrthoNets, this approach constrains the orthogonality within individual filters, effectively reducing the risk of model overfitting to some extent.

#### Orthogonality and its implications for neural networks.

The Special Orthogonal Group is a significant matrix group comprising orthogonal matrices over real numbers with determinants equal to 1. Formally, the n-dimensional Special Orthogonal Group SO(n) is defined as:

$$SO(n)=R\in {\mathbb{R}}^{n\times n}|R^{T}R=I,\det(R)=1$$ (1)

Where *R* is an n×n real matrix, ${\text{R}}^{\text{T}}$ is the transpose of *R*, *I* is the n×n identity matrix, and det(R) is the determinant of *R*. Matrices in *SO*(*n*) are orthogonal, satisfying *R*^*T*^*R* = *I*, ensuring their column (or row) vectors are mutually orthogonal and of unit length. This orthogonality guarantees that the column (or row) space forms an orthonormal basis. By leveraging *SO*(*n*)’s parameterized representation, manifold sampling, symmetry, and dimensional flexibility, this method obtain a set of orthogonal bases with strong orthogonality, good diversity, and adaptability to different task requirements. Utilizing orthogonal matrices generated from *SO*(*n*) as convolutional filters enhances feature representation quality. This approach ensures minimal redundancy and maximum independence between feature channels, enabling the network to capture richer and more diverse feature patterns. It also provides a regularization effect, mitigating overfitting and improving model generalization.

Consider a multi-layer network:

$$f(𝐱)=W^{\left(L\right)}\sigma (\cdots W^{\left(2\right)}\sigma (W^{\left(1\right)}𝐱))$$ (2)

Where σ(·) represents a 1-Lipschitz activation function, such as ReLU, and ${\text{W}}^{\left(\text{l}\right)}$ denotes the weight matrix or the unfolded convolutional kernel of the *l*-th layer. The function f(𝐱) represents the output of the deep neural network, with **x** as the input. Applying orthogonal constraints to some or all ${\text{W}}^{\left(\text{l}\right)}$, such that:

$$(W^{\left(l\right)}{)}^{T}W^{\left(l\right)}=𝐈,\;\|W^{\left(l\right)}{\|}_{2}=1$$ (3)

Where $\|\cdot {\|}_{2}$ denotes the spectral norm of the matrix, ensures the following property:

$$\|f(𝐱)-f(𝐲){\|}_{2}\leq \prod_{l=1}^{L}\|W^{\left(l\right)}{\|}_{2}\cdot \|𝐱-𝐲{\|}_{2}=\|𝐱-𝐲{\|}_{2}$$ (4)

Here, the Lipschitz constant of the entire network is $L_{f}=\prod_{l=1}^{L}\|W^{\left(l\right)}{\|}_{2}=1$. Under ideal conditions, where all layer weight matrices are strictly orthogonal, these layers do not amplify input perturbations. This helps maintain control over the overall Lipschitz constant of the network. Even when strict orthogonality cannot be achieved for all layers in practice, applying orthogonal constraints to critical layers and keeping their spectral norms $\|W^{\left(l\right)}{\|}_{2}$ within a small range (e.g., ≤C) effectively tightens the network’s overall Lipschitz constant. This constraint limits the network’s effective capacity, reducing the risk of overfitting.

### Improved efficient encoder for OS-DETR

**Fig 3:**
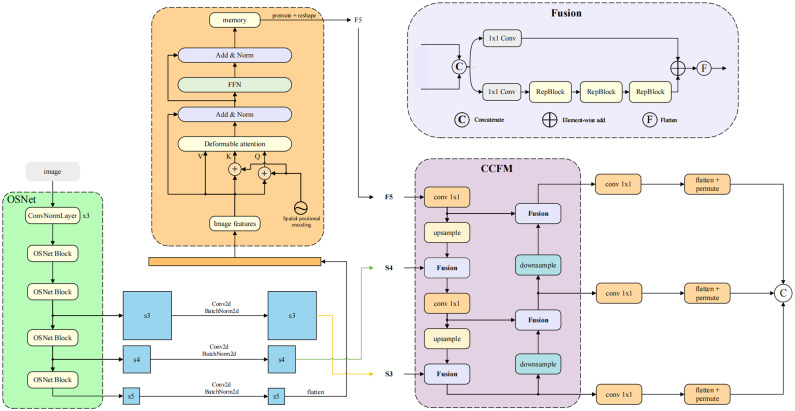
The structure of improved efficient encoder for OS-DETR Model. This encoder comprises an AIFI module and a CCFM module. The features extracted by the feature extraction network need to be dimensionally varied before they can be fed into the AIFI and CCFM modules.

In the encoder section of other multi-scale models, the extended input sequence length significantly impacts the computational efficiency, impeding the real-time capabilities of the DETR model. To address this challenge, this study introduced an improved efficient encoder, which structure is similar to the RT-DETR model. This encoder comprises two key components: the Attention-based Intra-scale Feature Interaction (AIFI) module and the CNN-based Cross-scale Feature-fusion Module (CCFM). The structure of this encoder is illustrated in Fig 3. This approach optimizes the processing of multi-scale features, enhancing both computational efficiency and model performance.

Unlike previous DETR models, such as Deformable-DETR [38], which typically convert feature maps from multiple scales into a lengthy vector, this study adopts the approach used in RT-DETR. It applies the AIFI module for within-scale feature interaction exclusively to the *S*5 feature plane extracted by the feature extractor. This strategy leverages the deeper, higher-level, and more semantically rich characteristics of *S*5 features compared to the shallower *S*3 and *S*4 features, making them crucial for the Transformer model. These features play a vital role in differentiating various object characteristics, as shallower features often lack rich semantic content.

In the AIFI module, the two-dimensional *S*5 features undergo initial flattening into a vector. They are then processed through deformable attention and Feed-Forward Network (FFN), before being reshaped back into two dimensions for subsequent cross-scale feature fusion using the CCFM module. The CCFM module comprises multiple fusion blocks with convolutional layers inserted into the fusion pathway. These blocks merge adjacent features into new ones, incorporating 3 RepBlocks. The outputs from both pathways are combined through element-wise addition.

This process can be described with the following mathematical expression:

$$Q=K=V=\text{Flatten}(S_{5})$$ (5)

$$\text{Out}=\text{CCFM}\left(\{S_{3},S_{4},\text{Reshape}(\text{DAT}(Q,K,V))\}\right)$$ (6)

**Fig 4:**
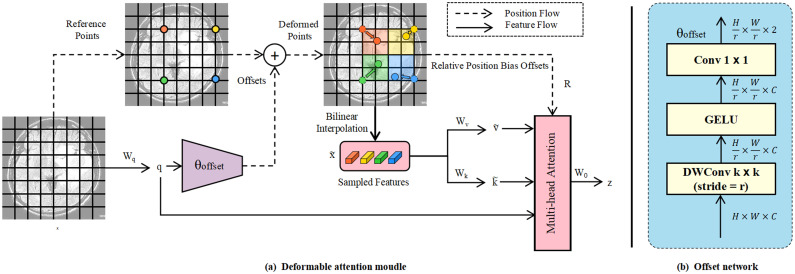
The structure of deformable attention.

The Reshape operation restores the feature’s shape to match that of *S*5, reversing the Flatten operation. Deformable attention [36] (DAT) is employed to address a limitation of the conventional multi-head self-attention mechanism used in models like RT-DETR. In the standard approach, the attention pattern remains fixed across all positions, with each attention head focusing on a weighted combination of all positions in the input sequence. These patterns do not change during training, which limits the model’s ability to capture complex relationships and dependencies within the data. Fig 4 illustrates the DAT structure. The deformable attention mechanism concentrates only on a select portion of key areas in the image, thus maintaining high performance while substantially reducing computational load. DAT dynamically chooses sampling points instead of uniformly processing the entire image, enabling the model to focus more on areas most relevant to the current task [46]. By learning a set of offsets for each input image, DAT allows all queries to interact with a common set of keys and values, shifting the key and value to crucial positions. DAT enhances the representation ability of sparse attention and also has linear spatial complexity.

Given an input feature map $x\in {\mathbb{R}}^{H\times W\times C}$, a uniform grid of points p is generated as references, which $p\in {\mathbb{R}}^{H_{G}\times W_{G}\times 2}$. Specifically, the grid size is downsampled from the input feature map size by a factor *r*, where *H*_*G*_ = *H*/*r* and *W*_*G*_ = *W*/*r*. The values of the reference points are linearly spaced 2D coordinates ranging from (0,0) to $\left(H_{G}-1,W_{G}-1\right)$. These coordinates are then normalized to the range [−1,+1] based on the grid dimensions $H_{G}\times W_{G}$, where (–1,–1) represents the top-left corner and (+1,+1) corresponds to the bottom-right corner. DAT first linearly maps the input feature map *x* to the query:

$$q=xW_{q}$$ (7)

Next, initialize a set of uniform grids as the reference points *p* for the sampling points, and the offset Δp is generated by the sub-network $\theta_{offset}$:

$$\Delta p=s\cdot tanh(\theta_{offset(q)})$$ (8)

Where *s* is the predefined control offset amplitude. Then, add the offset Δp to the reference point *p* to get the coordinates of the sampling points, and use bilinear interpolation to sample the original feature map *x*:

$$\tilde{x}=\Phi_{bilinear}(x;p+\Delta p)$$ (9)

Where $\Phi_{bilinear}(A;B)$ represents the bilinear sampling operation, the first operand *A* is the feature map, and the second operand *B* is the coordinates of the sampling points. The features are then sampled at the locations of the deformed points, serving as keys and values for the subsequent projection matrices. The deformed keys and values are obtained through the following expression:

$$\tilde{k}=\tilde{x}W_{k},\;\tilde{v}=\tilde{x}W_{v}$$ (10)

DAT employs a “Deformable relative position bias” method for position encoding, introducing spatial information into the visual attention mechanism. This approach encodes the relative position bias when considering the relative positions between queries and keys in the attention mechanism. Traditional attention mechanisms base the interaction between queries and keys solely on content similarity, neglecting their spatial relationships. To address this limitation, relative position bias is introduced to capture spatial information between queries and keys.

For a feature map of shape *H* × *W*, the relative coordinate displacements range from [–*H*,*H*] and [–*W*,*W*] in the two dimensions. In Swin Transformer [47], the authors construct a relative position bias table $\hat{B}\in R^{\left(2H-1)\times (2W-1\right)}$, indexing this table by relative displacements in both directions to obtain the relative position bias *B*. However, in deformable attention mechanisms, key positions are continuous rather than discrete. To accommodate this, the authors propose a number of improvements:

1. Continuous positions: Deformable attention allows keys to have continuous positions, not limited to discrete grid locations.
2. Normalized range: Relative displacements are normalized to the [−1,+1] range, enhancing robustness across different feature map scales.
3. Interpolation: Interpolation in the parameterized bias table covers all possible offset values, not just discrete relative displacements.
4. Parameterized bias table: A learnable parameterized bias table is used instead of fixed position encoding, allowing adaptive learning of position biases.

This method provides a flexible and adaptive approach to incorporating spatial information into the attention mechanism, particularly suited for deformable attention scenarios. The mathematical expression for this approach is as follows:

$$\tilde{B}=\emptyset_{\text{bilinear}}(\hat{B};R(p_{q},p_{\tilde{k}}))$$ (11)

Where $\hat{B}$ is a smaller-sized bias matrix, $R(p_{q},p_{\tilde{k}})$ is used to calculate the relative position of the query and the deformed key, and finally the output ${\text{z}}^{\text{m}}$ of each head in the multi-head attention is calculated as follows:

$$z^{\left(m\right)}=\text{softmax}\left(\frac{q^{\left(m\right)}{\tilde{k}}^{(m)⊤}}{\sqrt{d}}+\tilde{B}\right){\tilde{v}}^{\left(m\right)}$$ (12)

Where *d* is the dimension of the query and key, which is the same as in the regular Transformer.

### Decoder for OS-DETR model

The Decoder’s input comprises three essential components. The first part includes the content information of bounding boxes, which encapsulates the category data. The second part utilizes the coordinate information of bounding boxes, represented as 4D data (*x*,*y*,*w*,*h*), denoting the position and dimensions of each anchor. The (*x*,*y*) values indicate location, while (*w*,*h*) represent size and shape. The third input is the memory, which consists of multi-scale feature maps extracted from the image. The initial values for both the content and coordinate information of bounding boxes are derived from two sources. The primary source is a set of 300 queries selected using the IOU-aware query-selection method. Unlike the original DETR, where queries lack prior information leading to slower convergence, RT-DETR’s queries are chosen from the memory based on category confidence scores. The secondary source involves adding noise to ground truth data. The denoising approach aligns closely with the DN-DETR [48] model. It introduces noise to ground truth categories, which then serve as part of the Decoder input. Similarly, object coordinates undergo noise addition, contributing to the anchor coordinates in the Decoder input. The Decoder employs Multi-Scale Deformable-Attention for its attention mechanism. Its output encompasses both category predictions and bounding box coordinates. This design enables model to perform end-to-end learning directly from image pixels to bounding boxes and category predictions, eliminating the need for additional post-processing steps such as NMS.

### Intersection over Union (IoU) for OS-DETR

In object detection tasks, IoU [49] serves as a crucial metric for evaluating the precision of bounding box regression. IoU-based loss functions, such as GIoU, provide gradient signals for model training and accelerate convergence. DETR-based framework often employ IoU-related strategies to guide the generation of high-quality feature representations. For instance, the original DETR model utilizes GIoU and its associated IoU-aware query selection method. This strategy prioritizes matching predicted boxes with higher IoU to ground truth boxes during the training process’s matching algorithm. This matching results in a greater update magnitude for these high-quality predicted boxes during backpropagation.

However, standard IoU or GIoU may encounter challenges in accurately reflecting subtle discrepancies between predicted and ground truth boxes, particularly when the two boxes exhibit similar shapes but possess slight differences in size or position. To further enhance IoU’s discriminative capability, several improvement methods have been proposed. Inner-IoU [50] emphasizes the influence of the inner region of bounding boxes on the loss. It scales both the ground truth box and the predicted box by the same ratio, using their respective center points as references. It then computes the IoU using the scaled boxes, thus giving greater attention to the overlap of inner regions. By reinforcing the internal regions, this approach can accelerate bounding box regression and improve the model’s generalization performance.

Another significant improvement is MPDIoU [51], which addresses the challenge of distinguishing between predicted and ground truth boxes that share the same aspect ratio but differ in size. MPDIoU enhances discrimination by minimizing the distance between the top-left and bottom-right corner points of the predicted and ground truth boxes. It combines this distance information with IoU. MPDIoU forms the final metric by a weighted combination of the normalized corner point distance and IoU. MPDIoU demonstrates improved discrimination and optimization in bounding box regression tasks, particularly in distinguishing cases with similar positions but different sizes, or cases with close center points but insufficient overlap.

**Fig 5:**
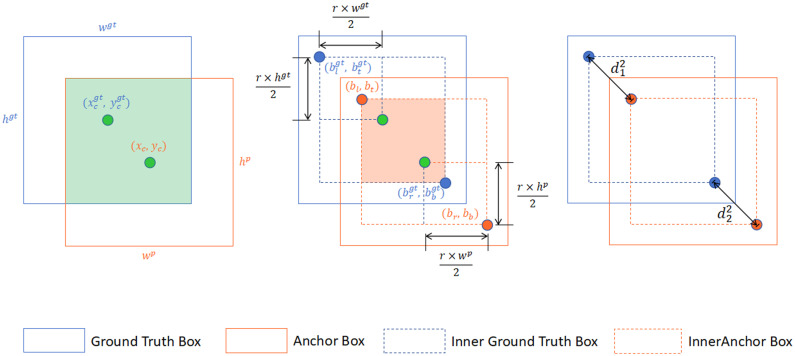
Description of IACD-IoU.

On this basis, OS-DETR integrates the strengths of Inner-IoU and MPDIoU, introducing the Interior-Aware Corner Distance IoU (IACD-IoU). This method simultaneously emphasizes the internal region influence of bounding boxes and the corner distance disparity. During IoU computation, a scaling factor is employed to highlight the overlap within the interior regions, followed by an additional penalty based on the distances between the top-left and bottom-right corners of the predicted and ground truth boxes. Specifically, the calculation of IACD-IoU can be seen as deriving an internally focused IoU, then subtracting a penalty term reflecting the corner distance, thus balancing fine-grained internal differences and global structural deviations, as illustrated in the Fig 5.

The method utilizes a scaling factor, denoted as *ratio*, set to 0.7 in this study, to compute the coordinates of the scaled ground truth and anchor boxes. The coordinates of the top-left and bottom-right corners of the ground truth box are represented as $\left(b_{l}^{\text{gt}},b_{t}^{\text{gt}}\right)$ and $\left(b_{r}^{\text{gt}},b_{b}^{\text{gt}}\right)$, respectively, while those of the anchor box are denoted as $\left(b_{l},b_{t}\right)$ and $\left(b_{r},b_{b}\right)$. The center of the ground truth box and its internal box is represented as $\left(x_{c}^{\text{gt}},y_{c}^{\text{gt}}\right)$, with its width and height expressed as $w^{\text{gt}}$ and $h^{\text{gt}}$. The scaled coordinates of the ground truth box are computed as follows:

$$b_{l}^{\text{gt}}=x_{c}^{\text{gt}}-\frac{w^{\text{gt}}\cdot \text{ratio}}{2},\;b_{r}^{\text{gt}}=x_{c}^{\text{gt}}+\frac{w^{\text{gt}}\cdot \text{ratio}}{2}$$ (13)

$$b_{t}^{\text{gt}}=y_{c}^{\text{gt}}-\frac{h^{\text{gt}}\cdot \text{ratio}}{2},\;b_{b}^{\text{gt}}=y_{c}^{\text{gt}}+\frac{h^{\text{gt}}\cdot \text{ratio}}{2}$$ (14)

Similarly, the center, width, and height of the anchor box are denoted as $\left(x_{c},y_{c}\right)$, $w^{\text{a}}$, and $h^{\text{a}}$. The scaled coordinates of the anchor box are given by:

$$b_{l}=x_{c}-\frac{w^{\text{a}}\cdot \text{ratio}}{2},\;b_{r}=x_{c}+\frac{w^{\text{a}}\cdot \text{ratio}}{2}$$ (15)

$$b_{t}=y_{c}-\frac{h^{\text{a}}\cdot \text{ratio}}{2},\;b_{b}=y_{c}+\frac{h^{\text{a}}\cdot \text{ratio}}{2}$$ (16)

The intersection and union of the scaled ground truth and anchor boxes are then calculated as follows:

$$\text{inter}=(\min(b_{r}^{\text{gt}},b_{r})-\max(b_{l}^{\text{gt}},b_{l}))\times (\min(b_{b}^{\text{gt}},b_{b})-\max(b_{t}^{\text{gt}},b_{t}))$$ (17)

$$\text{union}=(ratio\cdot w^{\text{gt}})\times (ratio\cdot h^{\text{gt}})+(ratio\cdot w^{\text{a}})\times (ratio\cdot h^{\text{a}})-\text{inter}$$ (18)

This results in the interior-focused IoU, denoted as ${\text{IoU}}_{\text{inner}}$:

$${\text{IoU}}_{\text{inner}}=\frac{\text{inter}}{\text{union}}$$ (19)

After computing ${\text{IoU}}_{\text{inner}}$, a penalty term is subtracted to account for the corner distance. The Euclidean distances between the corners of the ground truth and anchor boxes, $d_{1}^{2}$ and $d_{2}^{2}$, are defined as:

$$d_{1}^{2}=(b_{l}-b_{l}^{\text{gt}}{)}^{2}+(b_{t}-b_{t}^{\text{gt}}{)}^{2}$$ (20)

$$d_{2}^{2}=(b_{r}-b_{r}^{\text{gt}}{)}^{2}+(b_{b}-b_{b}^{\text{gt}}{)}^{2}$$ (21)

With *w* and *h* representing the width and height of the input image, the final form of IACD-IoU is given by:

$$\text{IACD-IoU}={\text{IoU}}_{\text{inner}}-\frac{d_{1}^{2}}{w^{2}+h^{2}}-\frac{d_{2}^{2}}{w^{2}+h^{2}}$$ (22)

## Experiments and results

### Dataset

**Fig 6:**
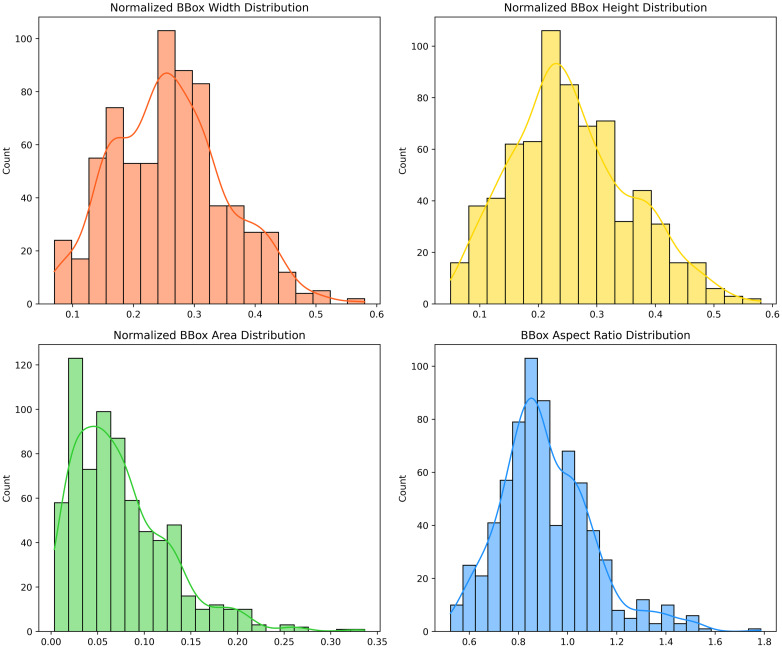
Distributions for brain tumor detection dataset.

This study employs the 2020 brain tumor detection dataset (Br35H) [52] for experiments. For a more objective evaluation, this study utilized the dataset provided by the RCS-YOLO project, comprising 701 images across train and val folders. Specifically, 500 images from the train folder were designated as the training set, and the remaining 201 images in the val folder served as the test set. Fig 6 shows the distribution details of the bounding boxes. The area of most bounding boxes is less than 20% of the total image size. This study further conducts a analysis of the performance of different orthogonality methods, using the Brain Tumor Dataset [53] as the experimental basis. The Brain Tumor Dataset is a simple dataset designed for brain tumor classification, comprising data samples from four categories: glioma, meningioma, no tumor (healthy brain), and pituitary.

### Experimental environment

This experiments were conducted on a system running Ubuntu 20.04, using Python 3.8 as the programming language and PyTorch 1.11.0 as the deep learning framework. The hardware setup consisted of an NVIDIA RTX 3090 GPU with 24 GB of memory.

### Evaluation metrics

This study employed a comprehensive and rigorous evaluation metric system to analyze the object detection performance of the model. At the same time, this study systematically evaluated the strengths and limitations of the proposed model. Specifically, the selected evaluation metrics encompass multiple aspects, including the model’s speed, complexity, and accuracy. Among them, FPS (frames per second) was used to measure the model’s real-time performance, and its calculation formula is as follows:

$$\text{FPS}=\frac{1000\;\text{ms (1 second)}}{T_{\text{pre}}+T_{\text{inf}}+T_{\text{post}}}$$ (23)

Here, $T_{\text{pre}}$, $T_{\text{inf}}$, $T_{\text{post}}$ represent the preprocessing time, inference time, and postprocessing time per frame, respectively. Each of these metrics is measured in milliseconds (ms). By calculating the total processing time across all stages and taking its reciprocal, the processing efficiency of the model in practical tasks can be determined. The number of parameters (measured in millions) and the GFLOPS (floating-point operations) indicate the computational complexity of the model. Precision and recall are used to evaluate the accuracy and completeness of the detection results, respectively. Their calculation formulas are as follows:

$$P=\frac{TP}{TP+FP},\;R=\frac{TP}{TP+FN}$$ (24)

Here, TP (True Positive) represents the number of correctly detected targets, FP (False Positive) refers to the number of incorrectly detected targets, and FN (False Negative) indicates the number of targets that were not detected. mAP@50 and mAP@50:95 denote the mean Average Precision at an IoU of 0.5 and the mean of a series of AP values calculated at IoU thresholds ranging from 0.5 to 0.95 with a step size of 0.05, respectively. These metrics comprehensively evaluate the model’s performance across different detection thresholds. IoU (Intersection over Union) is defined as the ratio of the intersection area to the union area of the predicted bounding box and the ground truth bounding box. The calculation of AP involves two steps: first, obtaining a series of Precision and Recall values under varying confidence thresholds to plot the Precision-Recall (P-R) curve; second, computing the area under the P-R curve to derive the AP. The mAP is obtained by calculating the arithmetic mean of the AP values across all categories, providing a comprehensive assessment of the model’s overall performance in multi-class detection tasks, ensuring the evaluation’s objectivity and thoroughness.

### Experimental parameter setting

The model underwent a training process spanning 100 epochs, utilizing a batch size of 4 and an image resolution of 640 pixels. The initial learning rate was set to 0.0001, while the final OneCycleLR learning rate reached 1.0. Early stopping patience was disabled to allow for comprehensive training. The training procedure employed 4 worker processes to optimize computational efficiency. Data augmentation techniques were strategically applied, with horizontal flipping implemented at a probability of 0.5. However, vertical flipping, Mosaic augmentation, and Mixup augmentation were not utilized in this particular training setup.

### Training results of OS-DETR

**Fig 7:**
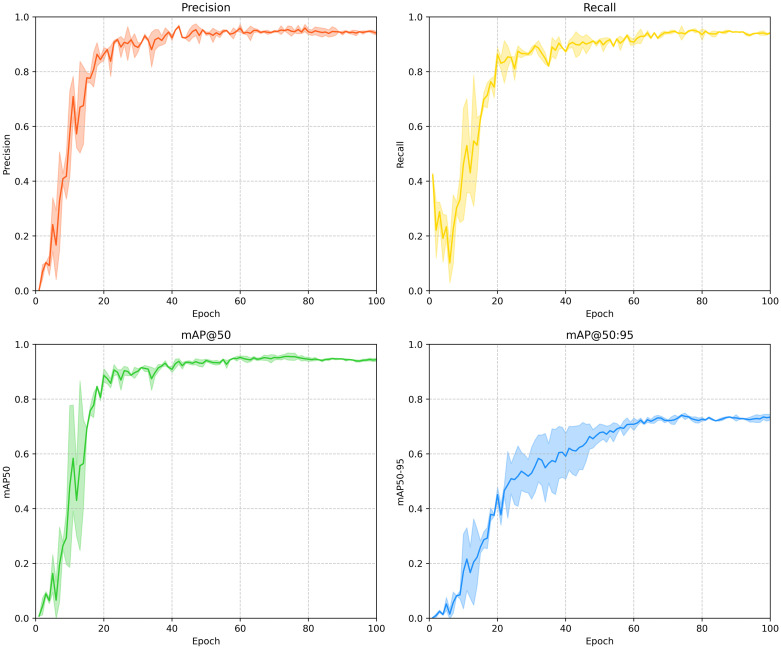
OS-DETR performance metrics across training epochs.

Fig 7 illustrates the performance metrics of OS-DETR under varying training epochs, including Precision, Recall, mAP@50, and mAP@50:95. Each subfigure presents a solid line representing the average experimental results and a semi-transparent shaded region indicating the standard deviation range calculated from multiple trials. The graphs reveal that all four metrics increase significantly during the initial training phase (the first 30 epochs), with the improvement rate slowing down after 40–60 epochs and eventually stabilizing. This trend suggests that the model undergoes rapid learning in the early stages, followed by a fine-tuning phase. Analyzing the error range shows substantial fluctuations in the early training phase, indicating sensitivity to initialization conditions and the order of training data. As training progresses, the error range gradually narrows, demonstrating enhanced stability in later stages. While YOLO-based framework typically require approximately 200 epochs to achieve convergence, the OS-DETR converges within 100 epochs, showcasing a notably faster convergence rate.

### Comparative experiments

#### Comparative experiments of orthogonality methods.

This section compares the performance differences between two orthogonalization methods: orthogonality among filters and orthogonality within filters. Specifically, experiments were conducted on the simple brain tumor classification dataset [53]. To minimize the influence of other modules, two classification models with simple structures were constructed based on standard convolutional neural networks. The only difference between the two models lies in the method of filter orthogonality employed.

**Fig 8:**
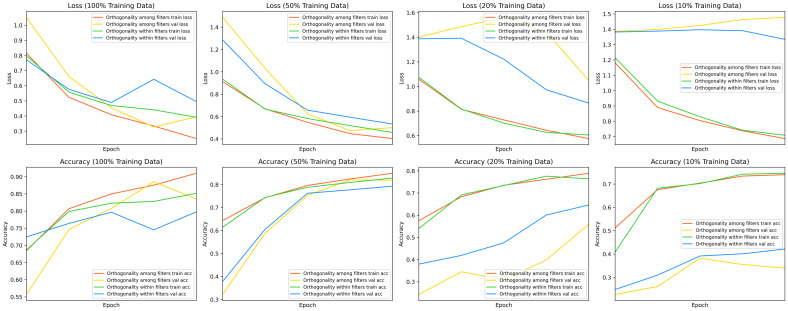
The comparison of different orthogonality methods at different training set proportions.

To evaluate the proposed OSNet, which reduce the risk of overfitting, especially in scenarios with limited data such as medical image, the two models were trained using training set proportions of 100%, 50%, 20%, and 10%. The training results are illustrated in Fig 8.

As shown in the figure, when the training set proportions were 100% and 50%, the Accuracy and Loss curves for both orthogonality methods exhibited similar trends, with some instances of overlap. However, as the training set size decreased to 20% and 10%, the model which using orthogonality within filters method demonstrated closer alignment of the Loss curves between the training and validation sets. Furthermore, its Accuracy curves exhibited greater consistency.

These results indicate that, in scenarios involving small-scale medical image datasets, the orthogonality within filters method effectively reduce the risk of overfitting. This validates the effectiveness of the proposed approach.

#### Comparative experiments on Br35h dataset

This study conducted comparative experiments on the Br35H dataset with other mainstream object detection algorithms (such as the YOLO series and DETR series models) and specialized brain tumor detection algorithms (such as BGF-YOLO, RCS-YOLO, and LSM-YOLO). All experiments were conducted on the same hardware to ensure consistency. To address inconsistencies in dataset preprocessing among different algorithms, the experiments utilized the dataset provided by the RCS-YOLO project for standardization. The experimental results are presented in Table 1.

**Table 1:** Comparison with YOLO-based models and other detectors.

Model	FPS↑	Params↓	GFLOPs↓	Precision↑	Recall↑	mAP_50_ ↑	mAP_50:95_ ↑
YOLOv5n	370	1.76	4.1	0.910	0.915	0.946	0.726
YOLOv5s	345	7.01	15.8	0.882	0.905	0.931	0.717
YOLOv5m	189	20.85	47.9	0.925	0.900	0.942	0.709
YOLOv5l	125	46.11	107.6	0.907	0.919	0.944	0.714
YOLOv5x	65	86.17	203.8	0.895	0.930	0.935	0.712
YOLOv7-tiny	167	6.07	13.0	0.940	0.930	0.946	0.733
YOLOv7	175	36.48	103.2	0.932	0.905	**0.960**	0.731
YOLOv7x	120	70.78	188.0	0.898	0.915	0.954	0.726
YOLOv8n	149	3.01	8.1	0.925	0.917	0.951	0.729
YOLOv8s	133	11.13	28.4	0.945	0.900	0.947	0.726
YOLOv8m	106	25.84	78.7	0.941	0.891	0.947	0.729
YOLOv8l	87	43.61	164.8	0.938	0.897	0.936	0.717
YOLOv8x	63	68.12	257.4	0.927	0.905	0.945	0.735
YOLOv9t	118	2.62	10.7	0.919	0.920	0.958	0.739
YOLOv9s	95	9.60	38.7	0.915	0.911	0.949	0.732
YOLOv9m	61	32.55	130.7	0.916	0.900	0.946	0.738
YOLOv9c	49	50.7	236.6	0.916	0.920	0.952	0.733
YOLOv9e	45	68.55	240.7	0.928	0.891	0.946	0.733
YOLOv10n	167	2.48	7.2	0.921	0.865	0.933	0.706
YOLOv10s	49	8.04	24.4	0.821	0.892	0.914	0.691
YOLOv10m	72	16.45	63.4	0.904	0.846	0.923	0.691
YOLOv10b	58	20.41	97.9	0.885	0.891	0.915	0.690
YOLOv10l	72	25.72	126.3	0.866	0.881	0.909	0.683
YOLOv10x	44	31.59	169.8	0.919	0.896	0.931	0.705
YOLO11n	84	2.60	6.3	**0.950**	0.896	0.949	0.724
YOLO11s	84	9.4	21.3	0.900	0.930	0.951	0.717
YOLO11m	78	20.0	67.6	0.912	0.924	0.948	0.735
YOLO11l	65	25.3	86.6	0.929	0.891	0.933	0.720
YOLO11x	53	56.8	194.4	0.901	0.902	0.946	0.716
DETR	11	36.74	101.4	0.842	0.767	0.912	0.646
DN-DETR	23	41.43	108.2	0.869	0.784	0.916	0.682
RT-DETR	111	20.18	58.6	0.938	0.925	0.942	0.728
RT-DETRv2s	48	20.08	60.4	0.864	0.826	0.927	0.714
RT-DETRv2m	38	36.40	99.8	0.874	0.823	0.928	0.719
RT-DETRv2l	32	42.70	136.3	0.870	0.824	0.931	0.717
RT-DETRv2x	24	76.37	259.1	0.861	0.847	0.931	0.729
BGF-YOLO [16]	65	3.39	22.2	0.922	0.940	**0.960**	0.726
RCS-YOLO [17]	77	45.70	94.5	0.941	**0.950**	0.943	0.737
RepVGG-GELAN [18]	41	25.24	102.4	0.949	0.896	0.948	0.732
LSM-YOLO [19]	101	2.87	12.4	0.924	0.925	0.955	0.735
**OS-DETR(ours)**	125	19.97	57.3	**0.950**	0.942	0.957	**0.742**

The results of the comparative experiments demonstrate that the proposed OS-DETR achieves significantly better performance than most mainstream YOLO and DETR series models in terms of precision, recall, mAP@50, and mAP@50:95 metrics. Even when compared with models specifically designed for brain tumor detection, OS-DETR exhibits superior performance. Specifically, OS-DETR outperforms RCS-YOLO, RepVGG-GELAN, and LSM-YOLO in the mAP@50 metric. Furthermore, OS-DETR achieves the best results in the mAP@50:95 metric, further confirming its outstanding performance.

Benefiting from the concise and efficient structure of OSNet, OS-DETR has fewer parameters and lower computational complexity than other DETR-based frameworks. Additionally, it surpasses all these DETR-based frameworks in terms of FPS, which highlights its high efficiency. Notably, some moedls suffer from extended post-processing time, causing the FPS of their smaller parameter versions to show limited improvement over larger parameter versions. Leveraging its end-to-end design, OS-DETR also achieves better FPS compared to YOLOv8m, YOLOv10b, YOLOv11m, and RT-DETR models with similar parameter scales.

#### Visualization of comparative experiments.

**Fig 9:**
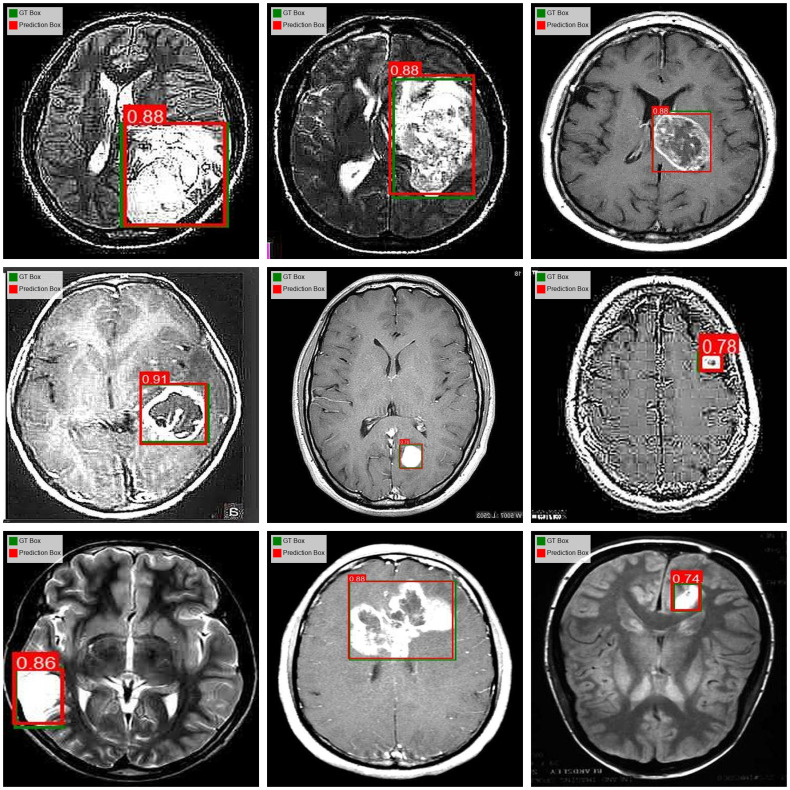
The comparison of OS-DETR predictions and the ground truth labels.

The comparison of the prediction results of the OS-DETR model on the validation dataset with the Ground Truth labels is shown in Fig 9. This figure presents a visual comparison between the model’s predictions and the ground truth annotations on several representative images from the validation dataset.

By comparing the prediction results of the OS-DETR model on the validation dataset with the ground truth labels, the results demonstrate that the OS-DETR model achieves outstanding accuracy in detecting brain tumors.

**Fig 10:**
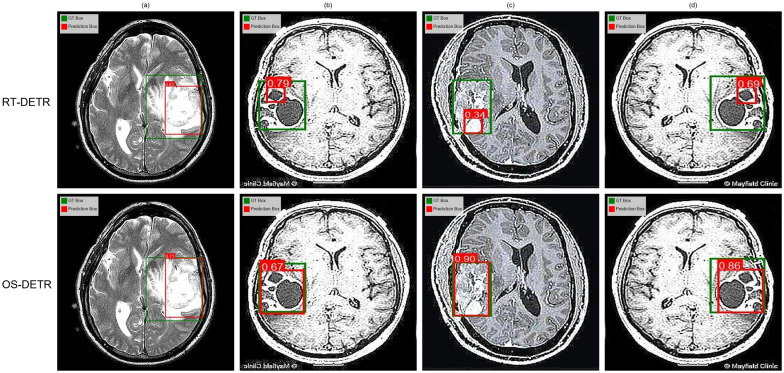
The comparison of OS-DETR and RT-DETR on challenging samples.

This study further compared the detection performance of OS-DETR and RT-DETR on challenging samples. As illustrated in Fig 10, in scenarios with blurred boundaries, such as cases (a) and (c), OS-DETR demonstrates superior capability in distinguishing targets from the background. In more complex scenarios, represented by cases (b) and (d) in Fig 10, RT-DETR primarily focuses on local target features, whereas OS-DETR exhibits a broader global perspective. This enables OS-DETR to generate more precise prediction boxes with higher alignment to the ground truth labels.

To investigate the regions of interest at different feature scales in the target detection task, this study applied the Grad-CAM++ method to visualize the multi-scale features of RT-DETR and OS-DETR. Specifically, two key layers from the backbone network were selected for analysis: layer 4, representing the first feature extraction stage, and layer 7, corresponding to the final feature extraction stage. Grad-CAM++ was used to compute activations and gradients during forward and backward propagation for these layers. The resulting activation heatmaps were normalized and overlaid on the original images to provide a more intuitive representation of the network’s focus areas.

**Fig 11:**
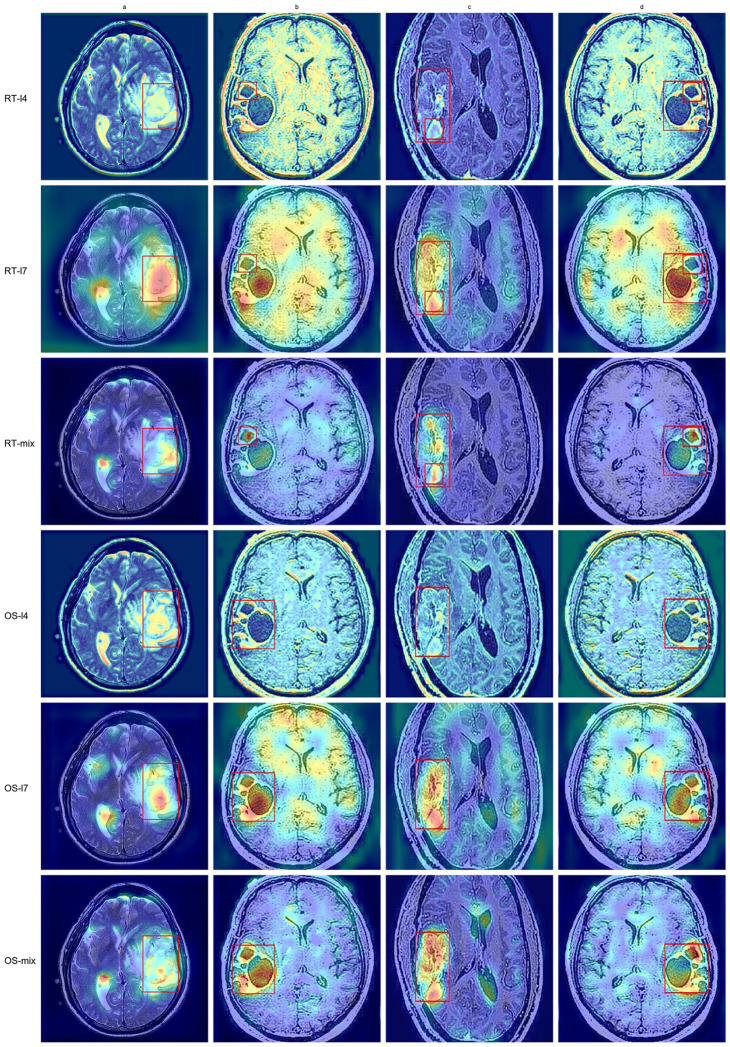
The comparison of heatmaps Between RT-DETR and OS-DETR.

Fig 11 illustrates the differences in the network’s focus at various layers for the same image. Features extracted at layer 4, after the initial Convolution Block and OSNet Block, primarily capture edges and local textures. In contrast, features from layer 7, processed through the final Convolution Block and OSNet Block, encapsulate higher-level semantic information, capturing more complete target representations.

To further analyze the effectiveness of multi-scale feature fusion in the detection head and neck, representative output layers were selected, and their fused outputs were calculated. By examining the generated heatmaps, the model’s focus across different feature layers and its capacity to perceive target regions were intuitively analyzed. This further demonstrates that RT-DETR primarily focuses on local target features, while OS-DETR exhibits a broader global perspective.

### Ablation experiment

To comprehensively evaluate the impact of key components in OS-DETR, a series of systematic ablation experiments were conducted, with the results detailed in Table 2. The results demonstrate that the integration of OSNet shows significant performance improvements across all evaluation metrics. Specifically, mAP@50 increased by 0.5%, while mAP@50:95 achieved a 1% improvement. When the enhanced efficient encoder with deformable attention mechanisms was incorporated independently, mAP@50 improved by 0.4%, and mAP@50:95 showed a 1.4% increase compared to the baseline model.

**Table 2:** Ablation experiment of OS-DETR.

OSNet	Improved Encoder	IACD-IoU	mAP_50_ ↑	mAP_50:95_ ↑
			0.942	0.728
✓			0.947	0.738
	✓		0.946	0.742
✓	✓		0.944	0.738
✓	✓	✓	0.957	0.742

Furthermore, when OSNet and the efficient encoder were integrated simultaneously, the model maintained its excellent performance. Notably, the introduction of ICAD-IoU further optimized the model, achieving the best overall performance. Compared to the original model, mAP@50 improved by 1.5%, and mAP@50:95 increased by 1.4%, reflecting a substantial enhancement in detection accuracy.

The combined effects of OSNet, deformable attention mechanisms, and ICAD-IoU significantly strengthened the target detection capabilities of the OS-DETR model. The incremental improvements achieved by each component highlight their complementarity and synergy, collectively driving continuous advancements in model performance.

## Discussion and conclusion

This study introduces an improved detection framework for brain tumor detection tasks in medical imaging, named OS-DETR (Orthogonal Channel Shuffle Detection Transformer). The framework incorporates OSNet as the backbone network and integrates deformable attention mechanisms along with an enhanced ICAD-IoU strategy within the DETR framework. OS-DETR demonstrates significant advantages in brain tumor detection tasks. Experimental results on the Br35H brain tumor dataset show that OS-DETR achieves superior performance across key metrics, with a Precision of 95.0%, Recall of 94.2%, mAP@50 of 95.7%, and mAP@50:95 of 74.2%, outperforming most mainstream object detection frameworks. This study highlights the potential of incorporating orthogonal characteristics and efficient encoding methods into deep learning models, offering a novel approach for small-sample object detection in medical imaging.

Although the proposed OS-DETR framework demonstrates significant advantages in brain tumor detection tasks, several areas require further research and optimization:

1. Although OS-DETR performs well on small-scale datasets, its computational cost still has room for further optimization. Future work could incorporate techniques such as network pruning, quantization, and distillation to design more lightweight models, addressing the constraints on computational resources in practical medical scenarios.
2. The current experiments are primarily conducted on the Br35H dataset. Future research could extend the application of OS-DETR to a broader range of medical imaging datasets, such as those involving different types of tumors or other diseases, to evaluate its generalization ability.
3. OS-DETR explores filter initialization based on the Special Orthogonal Group method. Future studies could investigate alternative methods for generating orthogonal matrices to further enhance model performance.
